# Electrochemical Behaviour and Galvanic Effects of Titanium Implants Coupled to Metallic Suprastructures in Artificial Saliva

**DOI:** 10.3390/ma11010171

**Published:** 2018-01-22

**Authors:** Ana Mellado-Valero, Anna Igual Muñoz, Virginia Guiñón Pina, Ma Fernanda Sola-Ruiz

**Affiliations:** 1 Clínica Dental Martínez y Mellado, S.L. Private Practice, 46930 Valencia, Spain; anamellado75@yahoo.es; 2 Institute for Industrial, Radiophysical and Environmental Safety, Universidad Politécnica de Valencia, P.O. Box 22012, 46071 Valencia, Spain; anna.igualmunoz@epfl.ch (A.I.M.); virgupi@iqn.upv.es (V.G.P.); 3 Prosthodontics and Occlusion Teaching Unit, Faculty of Medicine and Dentistry, University of Valencia, 46010 Valencia, Spain

**Keywords:** galvanic corrosion, ion release, dental alloys, implant supraestructures

## Abstract

The aim of the present study is to analyze the electrochemical behavior of five different dental alloys: two cobalt-chromium alloys (CoCr and CoCr-c), one nickel-chromium-titanium alloy (NiCrTi), one gold-palladium alloy (Au), and one titanium alloy (Ti6Al4V), and the galvanic effect when they are coupled to titanium implants (TiG2). It was carried out by electrochemical techniques (open circuit measurements, potentiodynamic curves and Zero-Resistance Ammetry) in artificial saliva (AS), with and without fluorides in different acidic conditions. The studied alloys are spontaneously passivated, but NiCrTi alloy has a very narrow passive domain and losses its passivity in presence of fluorides, so is not considered as a good option for implant superstructures. Variations of pH from 6.5 to 3 in artificial saliva do not change the electrochemical behavior of Ti, Ti6Al4V, and CoCr alloys, and couples, but when the pH of the artificial saliva is below 3.5 and the fluoride content is 1000 ppm Ti and Ti6Al4V starts actively dissolving, and CoCr-c superstructures coupled to Ti show acceleration of corrosion due to galvanic effects. Thus, NiCrTi is not recommended for implant superstructures because of risk of Ni ion release to the body, and fluorides should be avoided in acidic media because Ti, Ti6Al4V, and CoCr-c superstructures show galvanic corrosion. The best combinations are Ti/Ti6Al4V and Ti/CoCr as alternative of noble gold alloys.

## 1. Introduction

**Figure 1:**
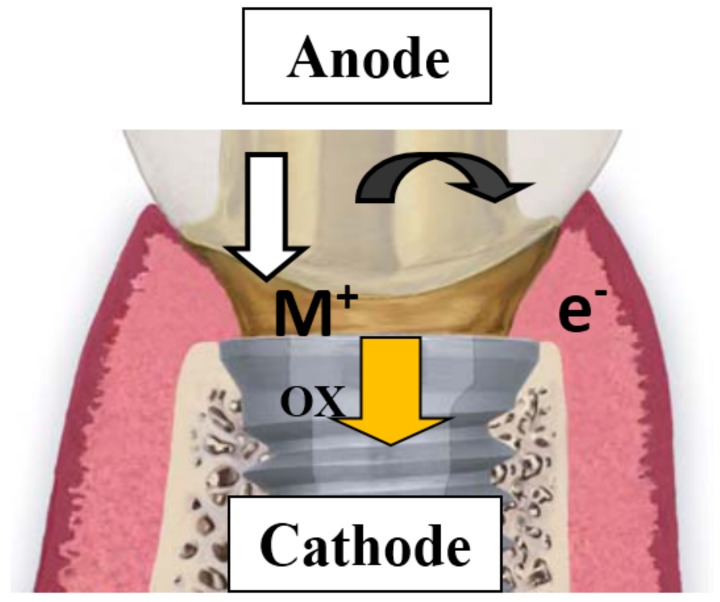
Galvanic couple formed in implants: the more active metal (less noble) acts as the anode releasing metal ions to the medium, while the less active metal (more noble) acts as the cathode.

Galvanic corrosion occurs when dissimilar alloys are placed in direct contact within the oral cavity or within the tissues. When saliva penetrates into prosthetic components in contact with implants, the metal dissolution generates currents, due to a potential difference created by the formation of a galvanic cell (Figure 1). In the case of dental implants, complicated electrochemical processes related to implant and suprastructure are linked to galvanic corrosion [1,2] which leads to a clinically relevant situation due to two main reasons: the biological effects that may result from the dissolution of alloy components, and the bone destruction caused by the current flow that results from galvanic coupling.

The alloy in the couple that corrodes would be the less noble or more active alloy. Coupling could result in an electropositive local environment along the implant interface, which could directly influence bone resorption. Hence, these galvanic couplings should be avoided [3].

Olmedo et al. (2003) [4] have observed that ionic release induced by corrosion could be responsible for peri-implantitis and treatment failure. Particles dissolved as a result of corrosion process are phagocytosized by macrophages (host response) and release mediators of inflammation in the form of cytokines (host defense), which inhibit the production of osteoblasts and promote osteolytic activity, leading to implant loosening. They found macrophages loaded with metal particles as indicators of the corrosion process in the soft peri-implant tissue of failed human dental implants.

The corrosion process may limit the metal’s resistance to fatigue, which may eventually cause the fracture of the implant. Guglielmotti and Cabrini (1997) [5] found metal particles included in the osseointegrated bone tissue of implants that failed due to metal fatigue, providing evidence of the corrosion of metallic structure.

The corrosion products can be distributed throughout the entire body, and may even cause allergic reactions (generally of the delayed type; Type IV) or a hypersensitivity reaction [2], with release of inflammatory mediators (cytokines). It has not been proved yet whether hypersensitivity to metal is the cause of implant failure or vice versa [6]. Metals like nickel, cobalt, beryllium, gold and palladium rank high in the allergy hit lists. Nickel leads the position on this list [7]. Therefore, the ultimate goal must be to use only those alloys with minimal ion release.

Commercially pure titanium (c.p. Ti) and its alloys have been widely used for dental implants due to their mechanical properties, good corrosion resistance in biological fluids and biocompatibility [8]. Although is widely accepted that titanium alloys are good materials for endo-osseous implantation [9], the choice of a suitable alloy for the suprastructure still remains an open question. Gold alloys are employed for suprastructures because of their excellent biocompatibility, corrosion resistance and mechanical properties. However, the increasing cost of precious alloys used in restorative dentistry has led to the development of low cost metallic materials for dental prostheses [3]. Major components of these alloys include nickel, cobalt or titanium. These alloys have good mechanical properties and are cost-effective, but their biocompatibility and corrosion resistance are of concern. It has been reported that failures of some implants was due to corrosion [4]. Thus, the design of suprastructures has to be made considering the corrosion resistance when the alloy is coupled to titanium, the biocompatibility, and the clinical studies of the relationship between the metal and the surrounding tissues (epithelium, connective tissue, and bone tissue) [10].

There are several factors such as the presence of fluoride ion, difference in oxygen concentration, dental plaque, microorganisms and mechanical stress that could increase further the corrosion rate, and should be also taken into account [11,12].

An overview of the existing knowledge on the galvanic corrosion mechanism in oral environment has been carried out through a literature search shown in Table 1, which summarizes the published studies on galvanic corrosion of dental implants/suprastructures.

Generally, the potential of the anode and the cathode is measured using a three-electrode configuration [10,13,14,15,16,17,18] and the galvanic effect quantified through the mixed potential theory. However, this approach has some limitations due to the initial hypothesis required for the application of the mixed potential theory [19]. Indeed, Al-Ali et al. [14] simulated the galvanic couple by welding different materials and they observed that the potential and current density of the welded couple did not correspond with the values obtained by the simple combination of the potentiodyinamic curves of the individual material. These values do not agree with experimentally obtained ones because the crevice corrosion is not taken into consideration in the simplified mixed theory, so it cannot be applied. Significant differences in corrosion behaviour were observed between welding joint and adhesive joint, finding by adhesive joining method higher values of coupled I_CORR_ than those for uncoupled alloys, and crevice associated with adhesive joint made the galvanic couple more corrosive than welding joint. Ciszewski et al. [15] also observed that the metal ion release determined through the galvanic current values obtained from accelerated electrochemical tests and applying the mixed potential theory were different from the direct measurements (adsorptive stripping voltammetry) of the metal ion release in the same couples. Indeed, metal ions from the cathode of the pair were also detected after 30 days of immersion.

To avoid the limitations of the mixed potential theory, specially obtained when studying passive materials, the galvanic effect was directly measured through a zero-resistance-ammeter (ZRA) [1,10,16,17,20,21]. Some other authors also tested the system superstructure/implant [18,22,23] and the measure of the corrosion damage was directly measured by quantifying the metal ion release after immersion tests of superstructures and implants couples by different analytical techniques such as the Inductively Coupled Plasma-Atomic Emission Spectrometry [23]. Yamazoe et al. [23] observed that titanium release in titanium/dental alloy is highly influenced by the titanium microstructure and in a titanium/titanium pair the metal ion release increases when differences in microstructure also increased.

In vivo studies suggest that polymetallism leads to corrosion process. Foti et al. [24] showed in animal models after 2 months, that the presence of a precious alloy superstructure leads to titanium migration towards the area around the cervical region of the implant, but without apparent modification of osseointegration, whereas with titanium superstructures this phenomenon was not observed. In humans, a wide range of galvanic currents resulted from electrical contact of metallic restorations in mouth has been noted. These currents are influenced by the area ratio, the total surface area of the galvanic couple, the particular conditions of each individual, chewing …. and the consequences were observed to vary depending on the location in the mouth, due to the degree of oral mucosa keratinization among others [25].

The aim of this study is to analyse the electrochemical behaviour in artificial saliva (AS) of cobalt-chromium alloys (CoCr), nickel-chromium-titanium (NiCrTi), gold-palladium alloy (Au) and titanium alloy (Ti6Al4V), used in the manufacture of the implant prosthesis structures and to evaluate the galvanic effect produced by the contact (physical or electrical through the electrolyte) of those with the commercially pure titanium grade 2 (TiG2). The influence of fluorides present in artificial saliva on the electrochemical processes and galvanic corrosion between dental implants and suprastructures are also studied.

The conclusions obtained were:

▪ Passive dissolution is the main corrosion mechanism of titanium and CoCr alloys and the corresponding passive dissolution rate was not accelerated by the presence of fluorides. NiCrTi alloy is the less corrosion resistant alloy among the studied materials in artificial saliva and fluorides critically accelerate its corrosion rate due to the susceptibility of nickel towards fluorides.
▪ Titanium alloys starts actively dissolving when the pH of the artificial saliva is below 3 and the fluoride content is 1000 ppm. Under these conditions, HF concentration is sufficiently high to form soluble titanium complexes in all studied titanium alloys.
▪ Measurement of galvanic corrosion of TiG2 implant coupled to different materials can only be carried out by zero-resistance ammeter and the direct measurement of the galvanic current and potential due to the passive nature of the biomedical alloys (CoCr, NiTiCr and Ti6Al4V).
▪ Acceleration of corrosion due to galvanic effects was only observed between titanium alloys and CoCr suprastructures in fluoride-containing acidic solutions. This galvanic effect is highly dependent on the solution chemistry and the coupled material, increasing when the suprastructure is Ti6Al4V.

## 2. Results

### 2.1. Open Circuit Potential (OCP)

**Figure 2:**
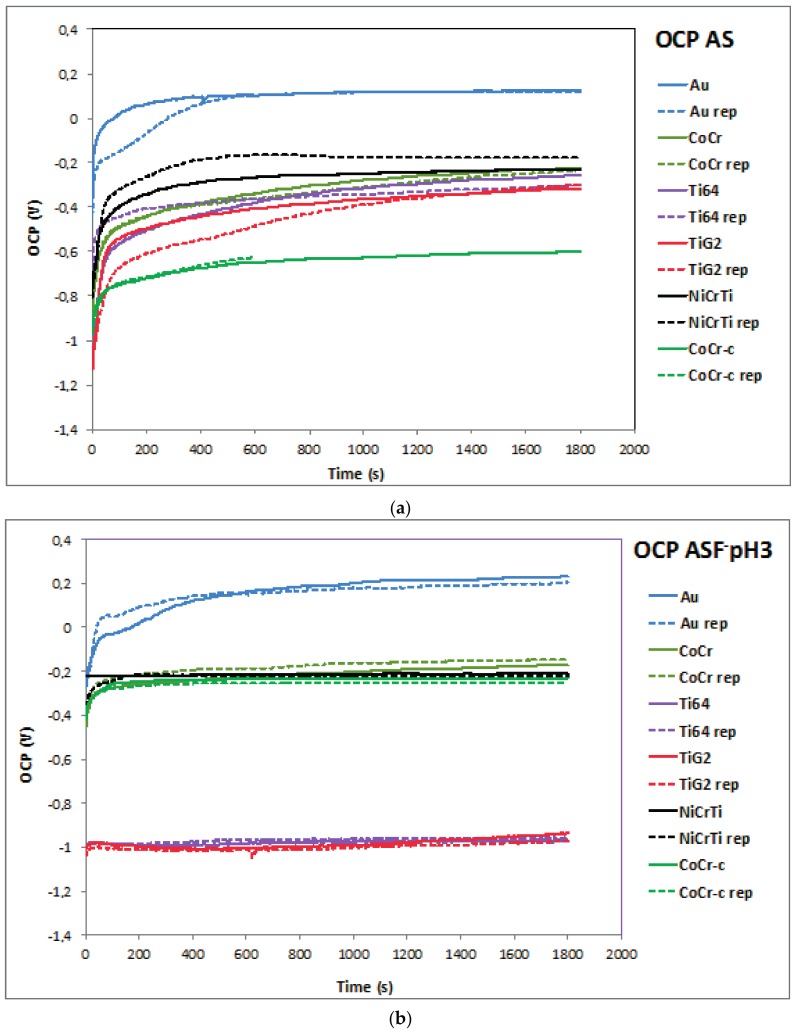
OCP evolution with time of the tested materials in (**a**) AS and (**b**) ASF^−^pH3. Legend: Au: gold-palladium alloy; CoCr: cobalt-chromium alloy; CoCr-c: cobalt-chromium cast alloy; NiCrTi: nickel-chromium-titanium alloy; Ti64: Titanium-6Aluminium-4Vanadium Titanium alloy); TiG2: Titanium grade 2; rep: repetition of the experiment.

OCP values of the tested materials were measured for 30 min in both electrolytes (Figure 2a,b). In all cases an abrupt increase of OCP was measured at the beginning of the OCP measurement due to the spontaneous formation of the passive films. The OCP stabilizes after 600 s and constant values were obtained at the end of the test. Those values are reported in Table 2.

The CoCr-c alloy exhibited the most negative potential (−611 mV) in AS while Au showed the highest one (121 mV). The other materials had intermediate OCP values, between −309 mV and −203 mV. In the fluoride content solution, Figure 2b, titanium alloys showed an intensive OCP decrease at around −1000 mV while the other tested materials established at OCP similar than the values in AS. Only the CoCr-c experienced an increase in OCP when immersed in ASF^−^pH3.

### 2.2. Potentiodynamic Curves

**Figure 3:**
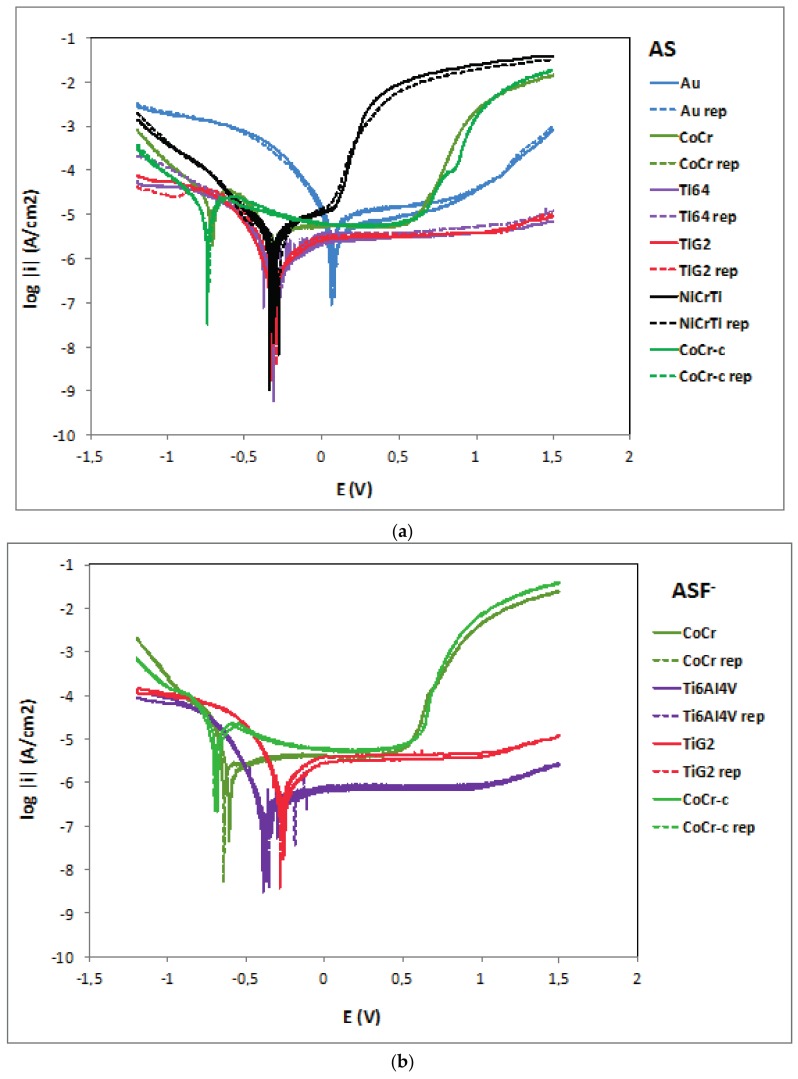

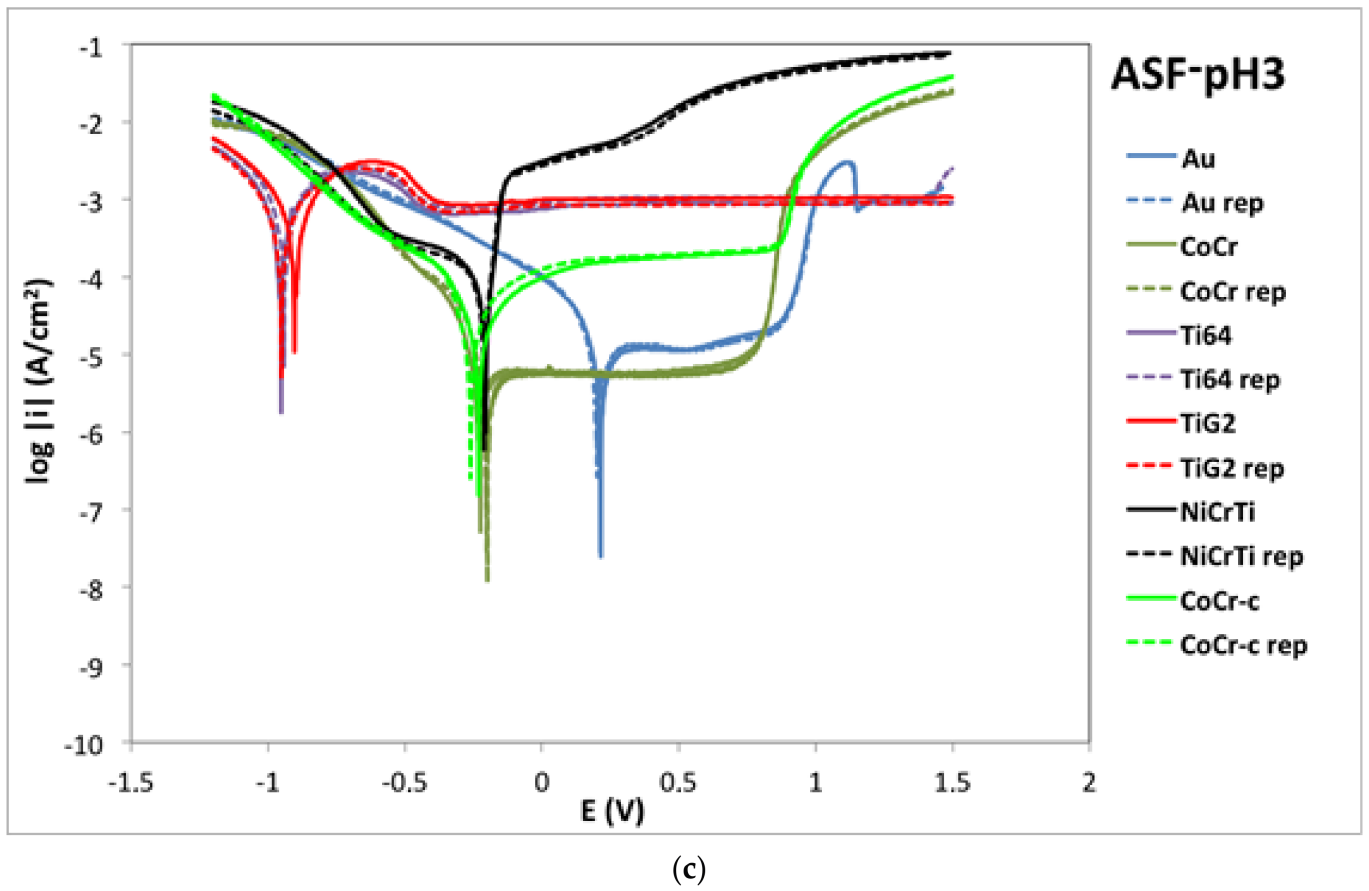
Potentiodynamic curves of the tested materials in AS (**a**), ASF^−^ (**b**) and ASF^−^pH3 (**c**). Legend: Au: gold-palladium alloy; CoCr: cobalt-chromium alloy; CoCr-c: cobalt-chromium cast alloy; NiCrTi: nickel-chromium-titanium alloy; Ti64: Titanium-6Aluminium-4Vanadium Titanium alloy); TiG2: Titanium grade 2; rep: repetition of the experiment.

Figure 3 shows the potentiodynamic curves of the studied materials in AS, ASF^−^ and ASF^−^pH3. In general, all polarization curves can be divided in four potential domains. In the cathodic domain the current density is due to the reduction of water and partially dissolved oxygen. The transition domain from cathodic to anodic current takes place at the corrosion potential (E_corr_), at which the corrosion current density (i_corr_) is obtained. The third domain corresponds to the passive zone due to oxide film formation on the metal surface and where the current density remains stable at the passive current density (i_p_). Finally, the tanspassive domain, corresponds to the zone comprised above the breakdown potential (E_b_), characterized by the increase in current due to the dissolution of the protective oxide film as well as water oxidation. Only titanium alloys do not show this transpassive region.

Electrochemical parameters obtained from the potentiodynamic curves for each material in electrolytes AS and ASF^−^pH3 are summarized in Table 3.

All the tested materials are metals and alloys spontaneously passivated in AS since their OCPs lied within their respective passive domains. In the acidic AS fluoride content solution (ASF^−^pH3) titanium alloys showed an active dissolution mechanism, thus presenting a significant decrease in the OCP and E_corr_, and an increase in i_corr_ and i_p_.

In AS, CoCr and CoCr-c alloys showed the lowest E_corr_ values (−738 mV) in good agreement with the lowest OCP values reported in the previous section. However, they presented a large passive domain. For potential values above 700 mV an increase in current density took place and began the dissolution of the oxide layer (chromium oxide). The NiCrTi alloy (E_corr_ −295 mV) shows higher E_corr_ than the CoCr alloys, but its passive domain is very narrow, showing transpassive dissolution at very low potential value (+158.50 mV) compared to the other materials. Au showed the highest E_corr_ and TiG2 and Ti6Al4V the broadest passive domain.

The i_corr_ of CoCr and NiCrTi are one order of magnitude higher than those obtained for the titanium and titanium alloy in AS, while in ASF^−^pH3, the NiCrTi and CoCr-c showed an increase of their i_corr,_ while both CoCr showed a decrease in its corrosion rate. Analogously, the i_p_ of the NiCrTi and CoCr-c in ASF^−^pH3 increases two orders of magnitude with respect to the values measured in AS. The CoCr and Au did not show any influence of the fluorides in its i_p_ (passive dissolution rate) although a decrease in E_b_ was observed.

In ASF^−^ (Figure 3b) the potentiodynamic curves of the studied materials are similar to those obtained in AS (Figure 3a), but produced a potential reduction in the passive domain of all materials and a slight increase in the current densities.

### 2.3. Zero Resistance Ammeter

**Figure 4:**
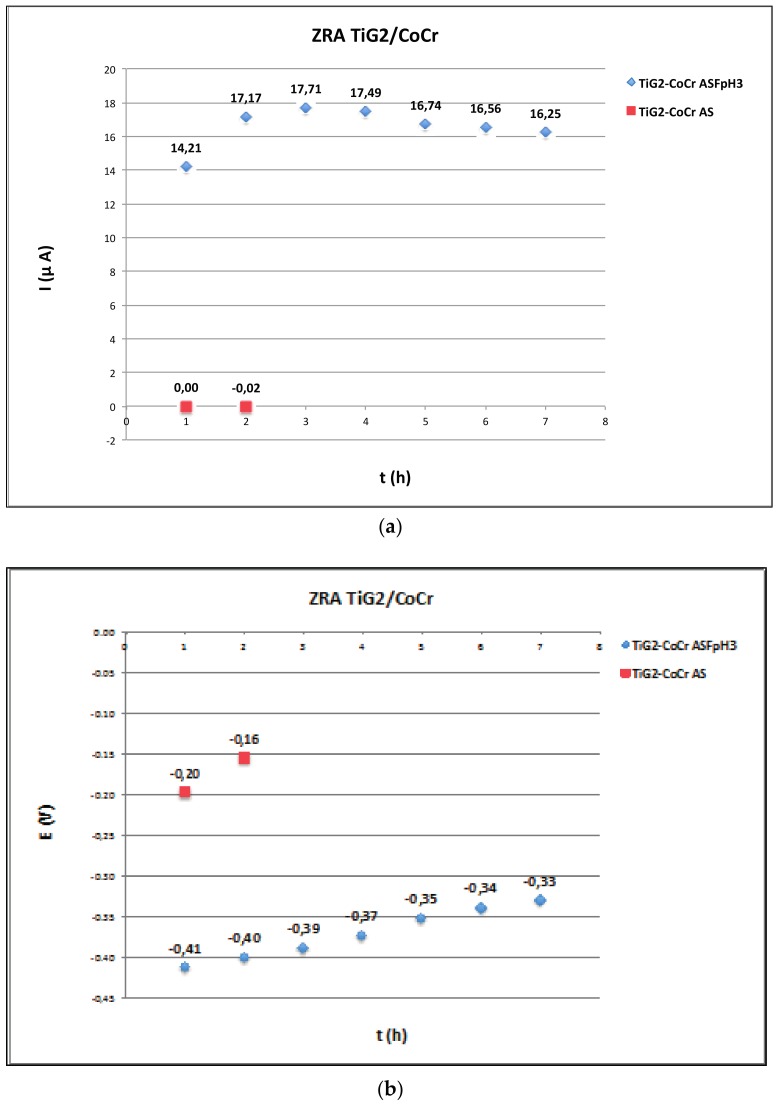
Average values at the end of every hour of immersion of the (**a**) galvanic current and (**b**) galvanic potential of the TiG2/CoCr pair in AS and ASF^−^pH3 as a function of time.

**Figure 5:**
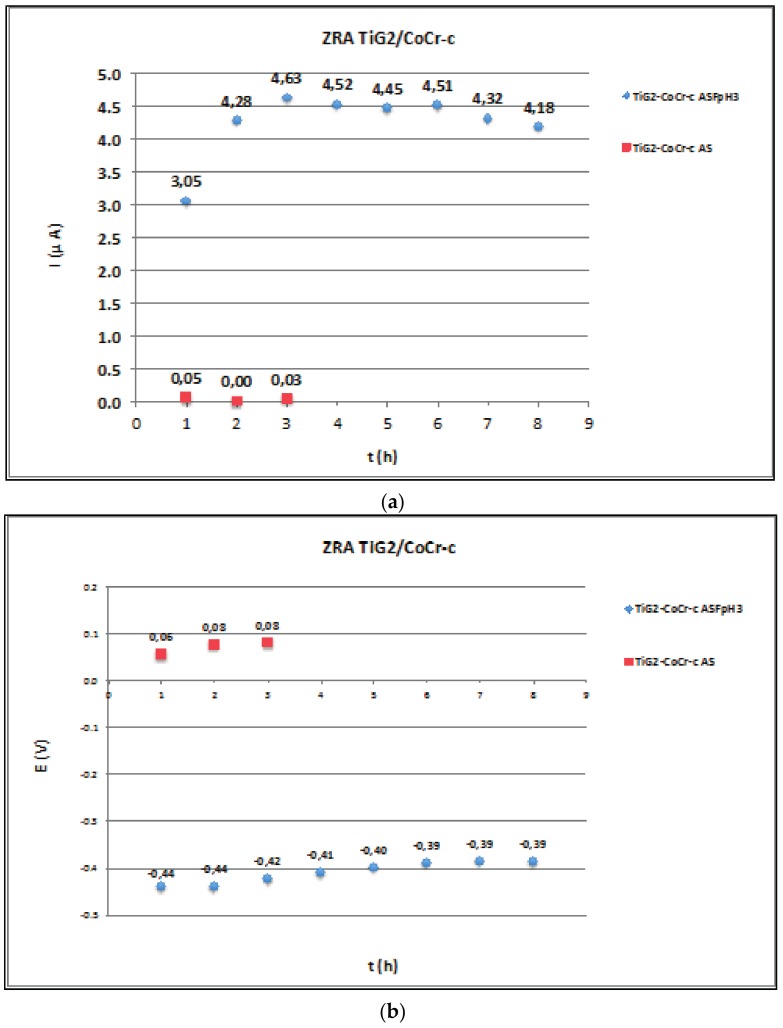
Average values at the end of every hour of immersion of the (**a**) galvanic current and (**b**) galvanic potential of the TiG2/CoCr-c pair in AS and ASF^−^pH3 as a function of time. Legend: Au: gold-palladium alloy; CoCr: cobalt-chromium alloy; CoCr-c: cobalt-chromium cast alloy; NiCrTi: nickel-chromium-titanium alloy; Ti64: Titanium-6Aluminium-4Vanadium Titanium alloy); TiG2: Titanium grade 2; rep: repetition of the experiment.

**Figure 6:**
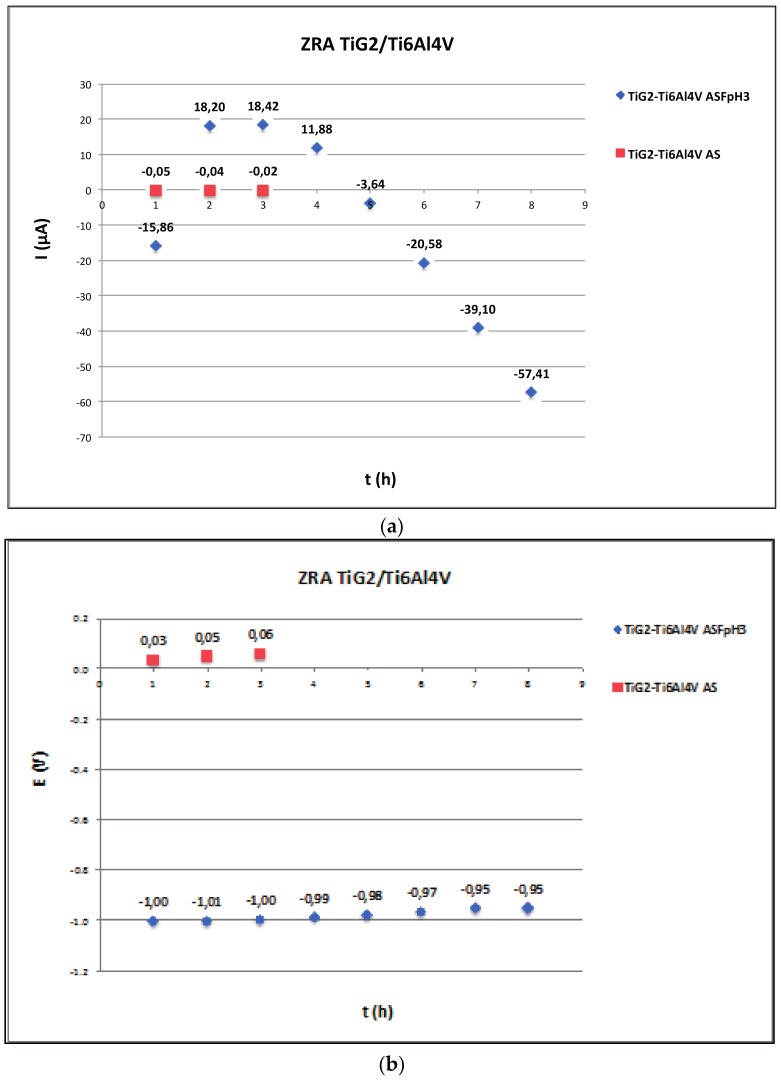
Average values at the end of every hour of immersion of the (**a**) galvanic current and (**b**) galvanic potential of the TiG2/Ti6Al4V pair in AS and ASF^−^pH3 as a function of time. Legend: Au: gold-palladium alloy; CoCr: cobalt-chromium alloy; CoCr-c: cobalt-chromium cast alloy; NiCrTi: nickel-chromium-titanium alloy; Ti64: Titanium-6Aluminium-4Vanadium Titanium alloy); TiG2: Titanium grade 2; rep: repetition of the experiment.

Figure 4, Figure 5 and Figure 6 show the average values at the end of every hour of immersion of the galvanic current and potential of the pairs TiG2/CoCr (Figure 4), TiG2/CoCr-c (Figure 5) and TiG2/Ti6Al4V (Figure 6) measured by Zero-Resistance ammeter.

The TiG2 coupled to both CoCr alloys in AS does not show any acceleration of its corrosion as a consequence of their galvanic coupling. Galvanic currents are very low and galvanic potentials remained constant around −200 and −400 mV for the TiG2/CoCr and TiG2/CoCr-c respectively. In the ASF^−^pH3 solution, the galvanic potentials decreased with respect to the values measured in AS, thus increasing the galvanic current, thus increasing the corrosion rate of the anodic member of the pair, the TiG2. Slightly higher galvanic current were obtained for the TiG2 in the TiG2/CoCr-c pair.

The TiG2/Ti6Al4V couple does not show any galvanic effect in AS but a huge increase in the corrosion rates in ASF^−^pH3. In the fluoride containing solution there are changes in the polarity of the pair depending on the immersion time. During the first hour and after 4 h of immersion Ti6Al4V alloy acted as the anode while it behaved as cathode between 2 and 4 h of immersion. The average galvanic currents of the pair were one order of magnitude than those measured in the other studied couples. Independently of the polarity of the pair, the galvanic potential remained constant at around −1000 mV.

## 3. Discussion

### 3.1. Corrosion Mechanisms

The Au alloy (60% Au, 30.6% Pd, 8.4% In) showed the more noble electrochemical behaviour among all studied materials according to their high OCP values (121.0 ± 7.1 mV), high E_corr_ (63 ± 15.6 mV) and the highest E_b_ (1195 ± 7.1 mV) when compared to the alternative alloys for the design of suprastructures such as CoCr, CoCr-c and NiCrTi. Those results are in good agreement with published data of noble alloys with gold and palladium [3,26,27]. Indeed, it has been previously reported in a study carried out on 29 noble alloys, 8 no noble alloys and 7 pure metals [27] that OCP values of the gold alloys (lying around +140 y +200 mV depending on the alloying elements) were very similar to those found for the pure metals (Au: 150 ± 1 mV y Pd: 139 ± 56 mV). Among the noble alloys studied by those authors, the Pd-base alloys showed higher corrosion resistance than the gold ones. This behaviour was justified by the presence of those noble metals in the alloy that gives heterogeneity to the structure and shifted the E_b_ of the alloy to higher values [27,28,29].

The corrosion mechanism of the titanium and cobalt alloys in the studied solutions is controlled by their passive dissolution. Passivity of the titanium alloys is imparted by the spontaneous formation of TiO_2_ [30] while Cr_2_O_3_ is the main compound in the passive film of CoCr alloy [31,32]. On the other hand, the NiCrTi alloy shows a very narrow passive region in AS and actively dissolves in the ASF^−^pH3 solution. Although chromium and titanium alloying are elements of the NiCrTi, thus promoting its passivity, Huang (2003) [33] observed that an amount of Ti less than 6% did not improve the corrosion resistance and the stability of the passive film in NiCr-based alloy. On the other hand, it contributes to the higher corrosion rate in acidic AS in presence of fluorides.

It has been previously demonstrated that a Cr content between 16% and 27% gives an optimal corrosion resistance to the alloy [21] and that the NiCrTi alloys that has a higher Cr content than 25% increase their corrosion resistance due to a more homogenous Cr distribution in the microstructure of the alloy [34]. Table 3 shows that NiCrTi has higher OCP and E_corr_ values than both CoCr aloys, however it shows the lowest E_b_ with a very narrow passive domain, due to the low Cr content of the NiCrTi (14.5%). Chromium oxide, as the main component of the oxide film and MoO_3_ in a less extent, increase the charge transfer resistance of the metallic ions through the passive film, while nickel oxides (Ni_3_O_4_/NiO) are more porous and showed less protective effect against corrosion. Nickel oxides are the second more concentrated compounds in the passive film of NiCrTi.

Electrochemical differences between CoCr and CoCr-c are due not only to the small differences in chemical composition but also to their fabrication process. The thermal process carried out during the casting and the impurities generated afterwards affect the final properties of the alloy [35]. Therefore, in AS, the CoCr-c showed worst electrochemical behaviour with lower OCP and E_corr_ and higher i_corr_ values than the CoCr, corresponding to the alloy with the less noble behaviour. However, the wide passive range due to its high Chromium content (>25%) and E_b_ above +790 mV, are well above the potential range registered in the oral cavity, which are around −300 y + 300 mV [36].

### 3.2. Influence of Fluoride Presence on the Corrosion Behaviour

With respect to the influence of fluorides in the corrosion mechanism, they do not modify the involved mechanism of the studied alloys at neutral pH. The spontaneous passivity in all the studied solutions can be observed by the OCP values, which do not significantly change in presence of fluorides. This suggests that fluoride ions did not hinder the formation of oxide layer on electrode surface even for high fluoride concentration [33].

However, the effect of fluorides at pH3 is more detrimental. On NiCrTi alloy it is observed a negative influence of fluorides on the electrochemical behaviour; due to the higher reactivity of nickel in presence of halides such as F^−^, which may cause possible clinical repercussions [37]. Previous studies on NiTi alloys already demonstrate the lower corrosion resistance of those alloys in presence of fluorides [38]. Indeed the TiO_2_-based surface film with trace amount of NiO was found to be more susceptible to fluoride-enhanced corrosion corrosion [39]. The presence of titanium on NiCrTi alloys does not provide higher passive dissolution resistance.

**Figure 7:**
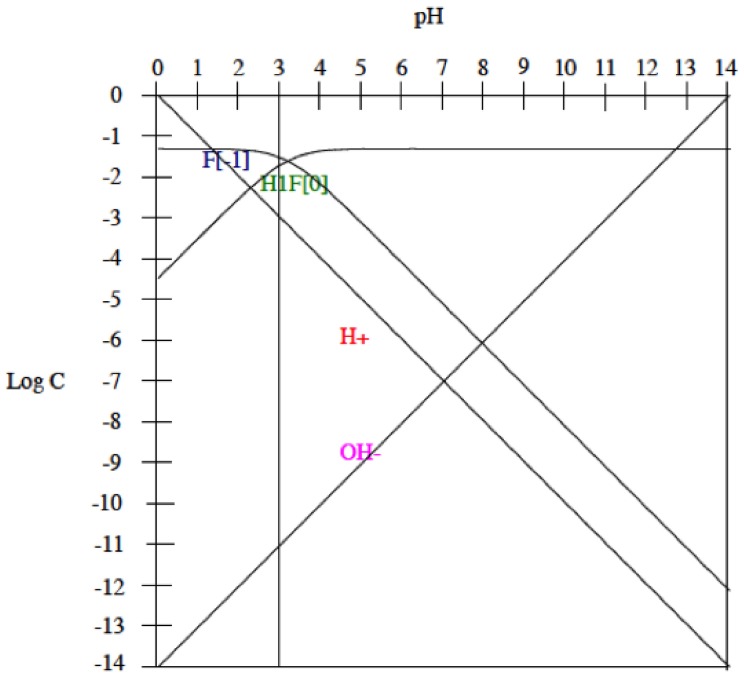
Equilibrium diagram of the species in the NaF solutions at different pH. Where C is the molar concentration of the species.

On titanium alloys fluorides content in the AS at pH 6.5 do not affect the corrosion rate, since 100 ppm of F^−^ can be considered a low concentration to be detrimental to the corrosion resistance of titanium or titanium alloys [9] but at pH3 the consequences are different. To analyse the effect of pH and fluorides on the titanium behaviour, the equilibrium diagrams of the fluoride species and their concentration at different pH according to the following equilibrium acidic constants (K_H_) of the fluorhidric acid are shown in Figure 7.

HF <---> H^+^ + F^−^

The assumption was made that the electrochemical reaction of fluorides on titanium is slower than the acid–base equilibrium. Consequently, the concentrations of the different fluoride species were supposed to be controlled by the acid–base equilibrium (expressed as dissociation reactions) and the corresponding acidic constant of deprotonation equilibrium is given in the following equation:

K_H_ = [H^+^] [F^−^]/[HF]

According to the equilibrium and in the ASF^−^pH3 the concentration of the involved species is, 1 × 10^−3^ M for [H^+^], 1 × 10^−11^ M for [OH^−^], 2.02 × 10^−2^ M for [F^−^] and 2.98 × 10^−2^ for [HF]. According to Nakagawa et al. [11] in acidic conditions, 30 ppm of HF could lead to the destruction of the passive film on titanium surfaces. Since, the pH of the electrolyte used in the present study is 3, the concentration of HF is higher (corresponds to around 60 ppm) than those 30 ppm. Kwon et al. [40] also confirmed that in those high HF concentrations the passive oxide layer on titanium alloys cannot remain stable.

**Figure 8:**
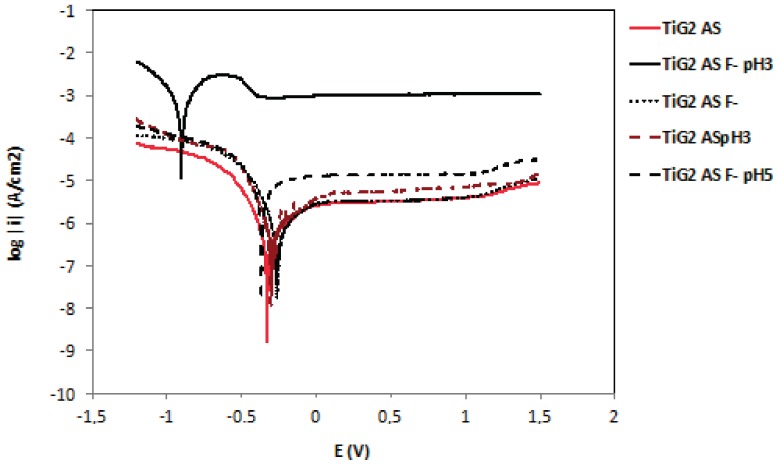
Potentiodynamic curves of TiG2 in different fluoride concentration medium and pH.

Indeed, TiG2 was tested in AS at different pH and fluoride concentrations and the potentiodynamic curves shows that the addition of 1000 ppm of fluoride ion without modifying the pH (6.5) has little influence on the electrochemical behavior of titanium. In the same way, a drop in pH to 3 in AS without fluorides does not register significant variations. But the combination of 1000 ppm of fluoride ion with the pH3 of the electrolyte causes a reduction in E_corr_ and increases the i_corr_ and i_p_, resulting in increased dissolution rates (Figure 8).

In the case of CoCr alloy, its corrosion resistance was improved in presence of fluorides as already reported by Takemoto et al. [41]. They observed a corrosion resistance of CoCr alloy in saline solution containing 0.1% sodium fluoride similar to the resistance shown by gold alloys.

### 3.3. Galvanic Corrosion

Galvanic corrosion between suprastructures and implants has been studied through the direct measurement of the galvanic current flowing between two different materials at the galvanic potential of the pair by zero-resistance ammeter (Figure 4, Figure 5 and Figure 6). Only net anodic current was measurable in the acidified solution in presence of fluorides, where TiG2 dissolved preferentially when coupled to both CoCr alloys. On the other hand, when TiG2 was coupled to the Ti64 alloy, a polarity change was observed. TiG2 acted as the anode of the pair at the beginning of the test but it converted into the cathode after 4 h of immersion. This change in polarity was registered in the two repetitions of the test carried out under the same conditions although the instant at which polarity was inverted varied between 3 and 4 h. In all cases, acceleration of Titanium dissolution is preceded by a potential shift towards lower values. When potential of the pair decreases, the galvanic current and thus the metal ion release increases. The decrease in potential can be due to the nature of the galvanic couple but also can be caused by the mechanical removal of the passive film during, for example, fretting conditions (i.e., fretting-corrosion conditions), which would lead to an acceleration of the corrosion rate of the material. Therefore, although the corrosion resistance of all the studied alloys is very high (Table 3) care has to be taken when coupling to a more noble material and the electrode potential shifts towards the active region of the alloy. On the other hand, titanium stability is extremely dependent on the solution chemistry and coupled material, generating a high metal ion released in presence of fluoride in an acidic solution and when coupled to another titanium alloy. In this last case, alloying with Vanadium and Aluminium decreases its corrosion resistance in long-term tests. This galvanic corrosion, resulting from coupling titanium with different metals or alloys, clearly increases the amount of released ions, which is a prerequisite for biologic effects to take place, and it has been reported as the one of the possible causes of implant failure after initial success [42].

## 4. Materials and Methods

### 4.1. Electrolytes and Materials

The solution employed to carry out the electrochemical tests is based on the the Fusayama artificial saliva (AS) which has the following composition: 0.4 g NaCl, 0.4 g KCl, 0.6 g CaCl_2_, 0.58 g Na_2_HPO_4_, 1 g urea and distilled water until 1 L. The pH of the AS solution was 6.5. Fluorides, 2.21 g/L of NaF (1000 ppm of fluorides), were added to the AS in order to analyse their influence on the corrosion behaviour of the metallic alloys (ASF^−^). The fluoride containing solution was prepared at pH 6.5 (ASF^−^) and pH 3 (ASF^−^pH3). Temperature of the solution was kept at 37 °C.

The selected alloys were provided by LAFITT (Valencia, Spain) and they correspond to materials commercially used for implants and suprastructures. They include two CoCr alloys, a NiCrTi alloy, a titanium alloy, one pure titanium grade 2, and a gold alloy. The chemical composition of the materials is given in Table 3 (the nomenclature of the materials used in the paper is given in brackets).

The materials were provided in form of metallic cylinders, which were embedded into non-conductive resin for the electrochemical tests thus leaving a working area of 0.28 cm^2^ in contact with the solution in the case of CoCr-c and NiCrTi alloys and of 0.5 cm^2^ for the rest of alloys.

Before each experiment, the samples were mechanically polished (500, 1000 and 4000 grit SiC paper), degreased with acetone, washed with pure water and dried with compressed air before use.

### 4.2. Electrochemical Equipment and Experiments

A double-wall three-electrode cell (volume 50 mL) in aerated conditions was used for all the electrochemical measurements. An Ag/AgCl 3 M KCl reference electrode and a platinum wire as counter electrode were used. All potentials were referred to the Ag/AgCl 3 M KCl electrode (205 mV versus SHE). All electrochemical measurements were carried out using a potentiostat Solartron 1287 (Solartron Group Ltd., Solatron Enterpraises, Torrance, CA, USA). Open Circuit-Potential (OCP) was measured for 30 min. The potentiodynamic polarization tests were performed by scanning the applied potential from −1200 mV and moved in the anodic direction to 1500 mV at a scan rate of 1 m Vs^−1^. The corrosion potential E_corr_ as well as the corrosion current density i_corr_ were automatically extracted from the polarization curves by the CorrView Version 3.0 software through Tafel slope extrapolation. Passivation current density (i_p_) and breakdown potential (E_b_) at a current density of 100 µA/cm^2^ were also obtained from the polarization curves.

Galvanic current and potential was measured as a function of immersion time using a zero-resistance ammeter (ZRA). TiG2 was coupled to the Ti6Al4V and both CoCr alloys and connected to the Solartron 1287 potentiostat used as a ZRA. The galvanic current and potential established between the pairs were measured every 0.5 s during several hours depending on the couple. The same reference electrode described previously was used. The current sign was positive when the direction of the electrons was from WE1 to WE2, thus WE1 was corroding. Current values were negative when the electrons flowed in the opposite direction, that is, the WE2 was corroding. The assays were designed with TiG2 as WE1 and the other alloys as WE2.

The reproducibility of the measurements was determined through three repetitions of each test.

## Figures and Tables

**Table 1:** Literature search about the existing knowledge on the galvanic corrosion mechanism in oral environment.

Ref.	Materials Implant // Suprastructure	Solution pH, T (°C)	Electrochem. Technique	Anal. Tech	Studied Parameters
Geis, 1989 [2]	c.p. Ti // NiCrMo y NiCrMoBe	Asa1 ^1^ 2.3	PD ^2^	AAS ^3^	V_corr_ (µg/cm^2^/día)
Lemons, 1992 [13]	Ti, Ti6Al4V, CoCrMo, 316L SS // Au alloys, Pd, Ni, Cu Amalgam	0.9% NaCl 7 ± 0.5, 37 ± 1	PD 2		E_corr_ (mV), i_corr_ (μA/cm^2^)
Venugopalan, 1998 [1]	Ti cp grade 2 // Au alloys, AgPd, 316L SS, CoCr, Ni (67%), Ni (70.4%) y Ni (77.5%) Amalgam	AS1 ^4^ without O_2_	OCP PD ^1^ (E_max_ = 1.2 V) ZRA		E (mV) vs. t (6 h), E_b_ (mV), E_M_ (mV) vs. t (6 h) I_g_ (μA/cm^2)^ vs. t (6 h), E_M_ (mV)
Reclaru, 1998 [10]	Ti c.p. grade 4 // Au alloys, Ag, Pd, CoCr y 316L SS	(1) ASFm ^5^ y ASFm^3^ + 0.1% F^−^ (without O_2_) (2) NaCl 1% y NaCl 1% + 0.1% F^−^ 6.15/5/4/3.5/3	OCP (24 h) PD ^1^ ZRA MPT^6^ Crevice corrosion (ASTM F746-81)	SEM ^7^	E (mV) vs. t (24 h), i (μA/cm^2^) vs. E (V) E_M_ (mV) vs. t (24 h), I_g_ (μA/cm^2^) vs. t (24 h)
Grosgogeat, 1999 [16]	Ti cp, Ti6Al4V // Au alloys, Ag, Pd, CoCr	(1) ASF-M ^8^: 5, 37 (2) AFNOR ^9^ with O_2_: 6.7, 37	-OCP (24 h) -PD ^1^ -ZRA (15 h) -TPM ^4^	SEM ^5^ AES ^10^	E (mV) vs. t (s), E_M_ (mV), i_g_ (nA/cm^2^)
Foti, 1999 [24]	Ti c.p // Ti, Au alloys	In Vivo		Histology IO ^11^	
Horasawa, 1999 [17]	Ti grade 2 // copper alloys and Gallium alloy	AS2 ^12^ 6.8, 37	OCP PD ^1^		E (mV) vs. t (s), E_M_ (V) i_g_ (A/cm^2^)
Cortada, 2000 [21]	Ti cp grade 1 // Ti cp grade 2, Ti cast cp grade 2, Au alloys, Pd, NiCr.	AS3 ^13^ without O_2_ 6.7, 37	OCP ZRA (250 min)	ICP-MS ^14^	E_z_, E_corr_, i_corr_
Taher, 2003 [20]	Ti cp grade 1 // Ter Ti ^15^, SSTi ^16^, Au, AgPd, NiCr, CoCr 1 (Co 61%-Cr 25%), CoCr 2 (Co 63.5%-Cr 30%) and amalgam	Asm ^17^ (ASTM, 1978) 7.2			E_M_ (mV) i_g_ (µA/cm^2^)
Oh, 2004 [18]	Ti cp grade 3 // Ti cp grade 3, Au alloy, AgPd, CoCr, NiCr	AS1 ^2^ 37	OCP (5000 s) PD ^1^, PS ^18^		i_p_ (µA/cm^2^) E_b_ (mV) i (μA/cm^2^) vs. E (mV)
Sutow, 2004 [25]	amalgam - amalgam amalgam - noble metal noble metal - noble metal amalgam - non-noble metal amalgam - noble metal - non-noble metal Groups: - News: ≤12 months - Old: >12 months	In vivo 35.1	-ZRA (15 s)		i-peak (µA) i-15 s (µA)
Al-Ali, 2005 [14]	Ti cp grade 2 // Au alloy, Au-Ag-Pt, Ag-Au-Pd -LW ^19^ Ti/noble alloy -MA ^20^ Ti/noble alloy	Ringer Solution	OCP (24 h), Rp (polarization resistance)		E_corr_ i_corr_ E_corr Mixto_ i_corr Mixto_
Ciszewski, 2007 [15]	- NiCr and CoCr alloys - Amalgam Combinations: - amalgam/NiCr - amalgam/CoCr -NiCr/CoCr	ASF ^21^ con O_2_ 5.6, 37	OCP (6 h) PD ^1^ EIS ^22^	VDA ^23^ (1, 2, 4, 6, 7 y 30 días)	E_corr_ (mV) i_corr_ (µA/cm^2^) i-peak (µA/cm^2^) i-15 s (µA/cm^2^) i-5000 s (µA/cm^2^)
Yamazoe, 2010 [23]	Ti cp and Ti6Al4V // Ti cp, Ti6Al4V, 5 aleaciones nobles MP ^24^, 5 aleaciones base Au, aleación Ag-Pd-Cu-Au, aleación base Ag -D I/S ^25^ -C I/S ^26^	Lactic acid 1%		ICPE ^27^ SCLM ^28^	

^1^ ASa: aireated solution C_3_H6O_3_ + NaCl + KSCN; 2 PD: Potentiodynamic curves; ^3^ AAS: atomic absortion spectrometry; 4 AS1: NaCl + KCl + NaH_2_PO_4_·2H_2_O + CaCl_2_H_2_O + Na_2_S·9H_2_O + urea; 5 ASFm Fusayama´s modified Artificial Saliva; ^6^ MPT: Mixed potential theory; ^7^ SEM: Scanning Electron Microscopy; ^8^ ASF-M: Artificial Saliva Fusayama-Meyer: NaCl + KCl + CaCl_2_·2H_2_O + NaH_2_PO_4_ + urea; ^9^ AFNOR: Carter-Brugirard (French Association of Normalization): NaCl + KCl + Na_2_HPO_4_ + NaHCO_3_ + KSCN + urea; ^10^ AES: Auger Electron Spectroscooy; ^11^ IO: Histomorfometric Analysis (osseointegration index); ^12^ AS2: Artificial saliva 2: KCl + NaHCO_3_ + NaH_2_PO_4_·H_2_O + KSCN; ^13^ AS3: Artificial Saliva 3: K_2_PO_4_ + KCl + KSCN + Na_2_PO_4_ + NaCl + NaHCO_3_ + urea; ^14^ ICP-MS: inductively coupled plasma-mass *spectrometry*; ^15^ Ter Ti: Ternary Ti (experimental:Ti-Ag-Cu); ^16^ SSTi: Reference material, Ti healing abutment; ^17^ ASm: Artificial Saliva modified, ASTM, 1978; ^18^ PS: Potentiostatic test; ^19^ LW: Laser welding; ^20^ MA: Mechanical alloying; ^21^ ASF: Artificial Saliva Fusayama: NaCl + KCl + CaCl_2_·2H_2_O + NaH_2_PO_4_·2H_2_O + Na_2_S·9H_2_O + NH_2_CONH_2_; ^22^ EIS: Electrochemical Impedance Spectroscopy; ^23^ Voltamperometría de Disolución Adsortiva.; ^24^ noble alloy for metal-porcelain restoration; ^25^ Direct union implant/suprastructure; ^26^ cemented union between suprastructures and implants; ^27^ Inductively Coupled Plasma-Atomic Emission Spectrometry; ^28^ scanning confocal laser microscope.

**Table 2:** Electrochemical parameters of the studied alloys in AS and ASF^−^pH3.

Alloy	OCP (mV)		E_corr_ (mV)		i_corr_ (µA/cm^2^)		i_p_ (µA/cm^2^)		E_b_ (mV)	
	AS	ASF^−^pH3	AS	ASF^−^pH3	AS	ASF^−^pH3	AS	ASF^−^pH3	AS	ASF^−^pH3
Au	121 ± 7	219 ± 18	63 ± 15	201 ± 1	1.7 ± 0.2	4.1 ± 0.7	12.2 ± 5	12.0 ± 0.1	1195 ± 7	940 ± 1
CoCr	−229 ± 9	−157 ± 16	−342 ± 1	−215 ± 17	1.3 ± 0.1	2.6 ± 0.4	5.3 ± 0.1	5.5 ± 0.1	790 ± 1	849 ± 1
CoCr-c	−611 ± 17	−237 ± 13	−738 ± 7	−246 ± 21	5.1 ± 0.5	10.3 ± 1.3	6.4 ± 0.1	189 ± 9	853 ± 6	890 ± 7
Ti6Al4V	−281 ± 31	−963 ± 9	−305 ± 4	−947 ± 7	0.1 ± 0.1	293.5 ± 67	3.3 ± 0.6	975 ± 114	-	-
TiG2	−309 ± 11	−954 ± 27	−311 ± 23	−925 ± 35	0.2 ± 0.1	238.0 ± 39	3.3 ± 0.3	940 ± 112	-	-
NiCrTi	−203 ± 39	−215 ± 6	−295 ± 22	−207 ± 3	1.2 ± 0.01	43.7 ± 6	12.1 ± 1	2921 ± 213	158 ± 10	290 ± 38

Legend: Au: gold-palladium alloy; CoCr: cobalt-chromium alloy; CoCr-c: cobalt-chromium cast alloy; NiCrTi: nickel-chromium-titanium alloy; Ti64: Titanium-6Aluminium-4Vanadium Titanium alloy); TiG2: Titanium grade 2; rep: repetition of the experiment.

**Table 3:** Chemical composition (wt %) of the studied alloys as given by the producer.

Elements	Alloys					
%wt	Ti Grade2 (TiG2)	Ti-6Al-4V (Ti6Al4V)	Co-Cr-Mo (CoCr)	Co-Cr-Mo Cast (CoCr-c)	Ni-Cr-Ti (NiCrTi)	Au-Pd (Au)
Ti	Bal.	Bal.	0.006		4	
Al		5.5–6.5	0.005			
V		3.5–4.5				
N	0.03	0.05	0.16			
C	0.1	0.08	0.036			
H	0.015	0.012				
Fe	0.3	0.25	0.07	<1		
O	0.25	0.13	0.01			
Co			65.32	59		
Cr			27.42	25.5	14.5	
Mo			5.51	5.5	9	
Mn			0.68			
Ni			0.07		72	
W			0.02	5		
Ga				3.2		1
Nb			LT 0.02	<1		
B			LT 0.01	<1		
Si			0.66	<1		
Au						60
Pd						30.6
In						8.4
Cu			0.01			
P			0.004			
S			0.002			
